# ‘There and back again’: revisiting the pathophysiological roles of human endogenous retroviruses in the post-genomic era

**DOI:** 10.1098/rstb.2012.0504

**Published:** 2013-09-19

**Authors:** Gkikas Magiorkinis, Robert Belshaw, Aris Katzourakis

**Affiliations:** 1 Department of Zoology, University of Oxford, Oxford OX1 3PS, UK; 2 School of Biomedical and Biological Sciences, Plymouth University, Plymouth PL4 8AA, UK

**Keywords:** endogenous retroviruses, HERV-K, pathogenesis, pathophysiology

## Abstract

Almost 8% of the human genome comprises endogenous retroviruses (ERVs). While they have been shown to cause specific pathologies in animals, such as cancer, their association with disease in humans remains controversial. The limited evidence is partly due to the physical and bioethical restrictions surrounding the study of transposons in humans, coupled with the major experimental and bioinformatics challenges surrounding the association of ERVs with disease in general. Two biotechnological landmarks of the past decade provide us with unprecedented research artillery: (i) the ultra-fine sequencing of the human genome and (ii) the emergence of high-throughput sequencing technologies. Here, we critically assemble research about potential pathologies of ERVs in humans. We argue that the time is right to revisit the long-standing questions of human ERV pathogenesis within a robust and carefully structured framework that makes full use of genomic sequence data. We also pose two thought-provoking research questions on potential pathophysiological roles of ERVs with respect to immune escape and regulation.

## 1. Introduction

Transposable elements (TEs) comprise almost half of the human genome. A significant proportion of them (almost 8% of the human genome) are the descendants of occasional germline invasions by exogenous retroviruses (XRVs) [1,2]. We call them endogenous retroviruses (ERVs) and they can be identified as DNA segments within animals' germline genomes that are similar to known retroviral sequences.

More than 10 years have passed since the first complete human genome sequence [3], and thousands of full human genome sequences have since become available [4]. One might think that we now have the technology to understand the role, if any, of ERVs in disease. However, mapping individual ERVs to their genomic positions remains a major experimental and biocomputing challenge, whereas studies of human ERV (HERV) expression are small with no clear consensus [5,6]. The fact that extensive research on HERVs has not shown clear pathologies, in contrast to ERVs in several other animals, suggests that research in HERVs still needs to be performed, but only within a rigorous and robust experimental framework. We argue that researchers have been ‘there’ (i.e. searched for potential pathogenic roles of ERVs) and failed likely owing to technological restrictions and fragmentary knowledge of cancer and autoimmunity, but now it is time to ‘go back again’.

Here, we summarize what we consider to be the most promising lines of ERV research that might illuminate the role of HERVs in pathophysiology. We emphasize the most important questions of the role of HERVs on human pathophysiology and describe some of the challenges that need to be tackled in order to have clearer understanding of HERVs' ecology. We start with a brief introduction to ERV taxonomy and the most important HERV families.

## 2. Classification of endogenous retroviruses

The classification system of ERVs divides them into three classes (I, II, III) [7]. If the XRVs were included based on their phylogeny, then class I would include the gammaretroviruses, II the betaretroviruses and III the spumaviruses. Well-defined groups within these classes determined by phylogenetic analysis are termed ‘families’. These families generally represent a single invasion followed by a copy-number expansion within the host's genome [8]. ERV families have traditionally been named after the amino acid carried by the tRNA complementary to the primer binding site (PBS) of the ERV genome. This nomenclature still holds for historical reasons, even if members of the same family do not have the same PBS complementary to the amino acid that names their family [9]. HERVs are classified into 31–40 families [10,11].

## 3. ‘Popular’ human endogenous retrovirus families

### (a) HERV-T: the typical

**Figure 1.**
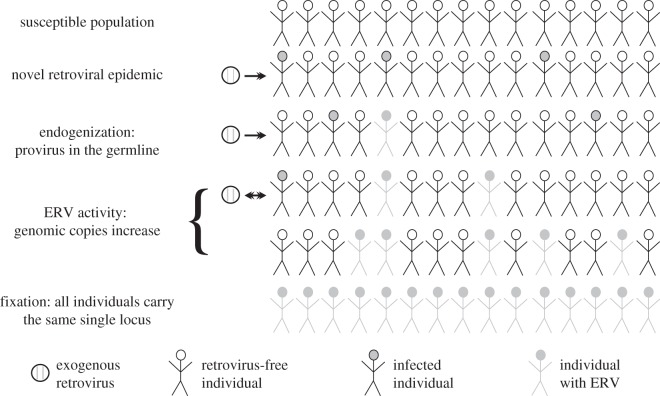
Population genetics of endogenous retroviruses. It has been suggested that ERVs that we currently identify in animal genomes are the result of ancient retroviral epidemics. Thus, the first step of ERV colonization involves the establishment of an epidemic in a susceptible population. Endogenization starts when retroviruses integrate into the host's germline and pass to the offspring through inheritance. The copies of the ERV in the germline constitute a family and may increase through time (ERV activity). Some of these copies become extinct or drift to fixation (i.e. every individual has the same locus).

HERV-T is a typical example of a small-to-medium-sized HERV family (approx. 60 copies or loci) in the human genome; others include HERV-S, HERV-K(HML5) and HERV-P. Most of these typical families have fewer than approximately 80 copies, an *env* gene and proliferate primarily by reinfection in the human genome (rather than retrotransposition or complementation) [12,13]. They have largely ceased generating new loci since approximately 35 Ma. Consistent with this observation is that the phylogenetic trees are star-like [14], indicating that ancient proliferation was followed by cessation of activity for tens of millions of years (figure 1).

### (b) HERV-L: the old

The oldest ERV family in the human genome is HERV-L. This family has members across a wide range of mammalian species, and is therefore believed to have infected the common ancestor of mammals [15]. The most striking characteristic of this ERV is that no one has ever found an *env* gene, meaning that it has been replicating through retrotransposition. ERV-L had bursts of replication in the mouse, the simian clade of primates and most recently in the elephant [15]. In humans, HERV-L ceased replicating approximately 30–40 Ma.

### (c) HERV-H: the abundant

The most abundant ERV family in humans is HERV-H, which accounts for about one-third of pol-containing ERV loci [9]. It invaded the primates about 30 Ma, around the separation of Old and New World monkeys [16,17]. Unlike HERV-L, there are HERV-H loci with *env* genes [18], which have been shown to be immunosuppressive [19]. It seems, however, that HERV-H replicated mostly through retrotransposition, with some re-infection [20] and trans-complementation [12].

### (d) HERV-W: the indispensable

This is a relatively small family that entered the genome of primates before the separation of Old and New World monkeys [21] and was active for only a short period, about 5 Myr [22]. This family, as well as HERV-FRD, have received increased research attention because their *env* genes have been co-opted (*syncytin*-1 and -2) to benefit their hosts [23,24]. HERV-W is also the only HERV family commonly copied by long interspersed nuclear elements (LINEs) [25].

### (e) HERV-K: the last (but not the least)

All but one of the HERV families have ceased replicating within the human genome. The family that might still expand, as evidenced by multiple human-specific insertions, and insertional polymorphism within the human population, is termed HERV-K (HML-2) [26–30], hereafter referred to as HK2. By using the consensus of different full-length HK2 loci, a functional infectious virus has been reconstructed *in vitro* [31,32]. HK2 is the only family that has loci with all of their open reading frames (ORFs) intact [27,30,33–35], compared with the rest of the families that have been inactivated with frameshifting indels and premature stop codons (see §4).

## 4. Human endogenous retroviruses are inactivated, downregulated or become replication defective through random knock-out mutations, hypermutation and silencing mechanisms

Most HERV loci are replication defective owing to mutations acquired during host germline cell division, which can cause premature stop codons [2] or frameshifts. The exception to this rule are those ERV genes that have been co-opted to provide a functional role to the host, and thus maintain coding capacity owing to purifying selection (e.g. *syncytin*-1) [36]. Another exception is some HK2 loci, which are very recent integrations [30]; even if HK2 loci have intact ORFs, they are transcriptionally and translationally inactive or replication defective. This can result from various mechanisms, including histone and DNA methylation [37,38], antiretroviral hypermutation [39,40] and potentially through RNA silencing as in other retrotransposons [41–44].

## 5. Criticism against the possible role of human endogenous retroviruses and human disease

The role of HERVs in human disease has been investigated for at least three decades. XRVs are often oncogenic, and the initially high expectation to find that HERVs were a cause of human cancer [45,46] or autoimmunity was followed by disappointment and dead-end projects. These unsuccessful attempts gave rise to the term ‘rumour viruses’ as a description of ERVs [47] (a play on the term ‘tumour viruses’) to reflect the lack of evidence of a pathogenic effect.

Before the deciphering of the human genome, our knowledge of HERVs was fragmentary [3,8,11], but since then, as well as the availability of many more animal genomes, the behaviour of ERVs and other TEs as genomic parasites is being clarified [48]. High-throughput sequencing provides an opportunity to study the mobility of these viruses in populations and individuals. For example, it has recently been shown using high-throughput sequencing [49] that *L*1 and *Alu* retrotransposons are copying within the brain during development [50,51], though at low levels [52], and within cancer cells [53]. We also know much more about the pathogenesis of cancer and autoimmunity. These advances now allow us to build more solid hypotheses about the role of HERVs in the development of disease than we were able to 10 years ago, as well as test them more rigorously.

ERVs may be involved in pathophysiological mechanisms either through their replication or through expressed gene products. Another potential mechanism would be through ectopic recombination between HERV copies (as it is for other genomic repeats) [54,55], but we will not analyse this in depth here. First, we are going to discuss the replication cycle as a possible pathogenic mechanism operating in the most recently active HERV, HK2. Second, we are going to examine the possible role of *env* genes in human pathophysiology.

## 6. The search for a mobile human endogenous retrovirus: HK2

### (a) Is HK2 active in the human germline?

**Figure 2.**
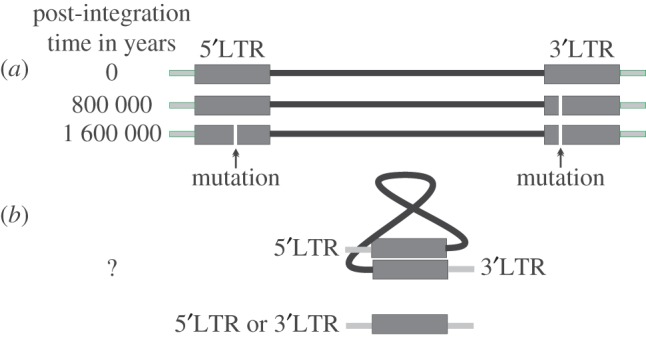
The fate of ERV long terminal repeats (LTRs). When a retrovirus integrates in the host's genome (time 0), LTRs are identical. Over time, they accumulate mutations at a host's substitution rate; thus the divergence of the LTRs from the same locus can be used to estimate how much time has passed since integration. On many occasions, a host genomic repair mechanism uses LTRs from a locus as a template to loop-out the internal region of the ERV resulting in remnant LTRs known as solo-LTRs. (Online version in colour.)

As we mentioned in §4, HK2 is the only HERV family that has definitely been copying itself after the human and chimpanzee split [26–28,56]. This is proved by multiple human-specific HK2 loci for which LTR-dating supports integration times at least as recent as 1 Ma (figure 2) [57], and by the existence of insertionally polymorphic loci within humans [28]. The unanswered question is whether HK2 contains replication competent loci and still increases its copy number within our genome (germline activity).

Two HK2 loci (K106 and K115) have identical LTRs [58], suggesting that HK2 was producing new germline insertions around (or earlier than) 0.8 Ma, assuming a divergence rate of 0.13% per Myr [56]. However, mutations accumulate stochastically at this rate, and a locus could have identical LTRs but be much older (or younger). Another dating method [59] uses a sample of the 5′ LTR sequences of a specific locus in the population and estimates the coalescent dates of these LTRs. These authors suggested that the convergence date of a population sample of K106 LTRs is between 0.09 and 0.15 Ma, which is after the emergence of anatomically modern humans [58]. Finally, data assuming a neutral model of HERV insertions indicate that the insertional polymorphism data of HK2 are consistent with activity continuing up to any time between the present day and 0.5 Ma [28].

Another group has found HK2 loci in fossil archaic human remains (a Denisovan) that are not in the modern human genome, suggesting that HK2 was active after the modern human–Denisovan split (approx. 0.8 Ma) [60]. However, finding loci in the Denisovan genome, which are not in the modern human reference genome, is not definitive proof of active infections after the human–Denisovan split. Two other processes could result in Denisovan-specific loci. First, at the time of Denisovan–modern human divergence, many HK2 loci were presumably unfixed within the common ancestral population; therefore some will have gone to fixation in the Denisovan and been lost from the human population. A similar process has led to a HERV-K locus being present in the same genomic location in gorillas and chimpanzees but not in humans [61]. Second, the human reference genome contains mostly fixed HK2 loci and only a minority of unfixed ones. This is because the reference genome (GRCh37) is the consensus of 13 genomes [62] and as such is likely to contain only fixed or very common loci. We expect that at least some polymorphic loci in the modern human population have not been mapped (e.g. some were identified in [63]), so it is probable that some loci of the Denisovan genome would have unfixed orthologous loci in the human population.

If we conservatively summarize the above, then our best estimate of HK2 copying activity in the human genome is that it was active more recently than 0.8 Ma with the most recent integration that went to fixation being around 0.1 Ma. To improve this estimate, we need to map the polymorphic loci in a large sample of humans to test the hypothesis that HK2 is still active or ceased replication 0.1–0.8 Ma using modelling based on population genetics theory [28]. Both mapping polymorphic loci and simulating the human population during the last 1 Myr are extremely challenging. Furthermore, an approach based purely on population genetics cannot definitively prove or reject a very recent (e.g. within the past 0.02 Myr) cessation of activity. If HK2 is actively replicating as a lineage, then there is a substantial probability that it would have pathogenic potential as has been shown for active families in other animals, because replication cannot be decoupled from insertional mutagenesis [2,64]. We note, however, that because HK2 is a family that invaded the genome at least 30 Ma, we do not expect high virulence (i.e. it should not significantly increase mortality and morbidity during the first 30 years of a human's life).

### (b) Theoretical evolution suggests that human endogenous retroviruses could be pathogenic at the post-reproductive age of the host

HK2 is an old family within which is a small branch that contains some recently integrated loci with largely intact *env* genes. The existence of multiple HK2 loci in human genomes means that these loci cannot be strongly deleterious, at least not prior to reproductive age [65]. Population genetics theory predicts that a slightly harmful allele can drift to fixation, especially if the pathogenicity is mainly expressed after the age of reproduction in the same way as a trait associated with senescence [66–75]. HK2 loci could have slight pathogenic potential especially at the post-reproductive age, which also coincides with the higher incidence of autoimmunity and cancer.

### (c) HK2 is upregulated in patients with cancer and other diseases

The observation that cancer cells produce virus-like antigens and particles is very old [76]; however, it is a phenomenon that has only partially been described and its role in cancer biology is still under investigation [77] (see §5).

Since the sequencing of the human genome in 2001 [3], more rigorous studies on the upregulation of HERVs in health and disease have been undertaken [78–87]. HK2 RNA has been found most frequently in the plasma of patients with HIV-1, breast cancer and lymphoma, with titres up to 10^7^ copies ml^−1^ [82,85]. Only rarely and with much lower titres has it been found in patients with rheumatoid arthritis, HCV and/or normal volunteers [85]. Viral and virus-like particles have been found at a fraction of 1.16 g ml^−1^ using electronic microscopy of plasma from patients with breast cancer and HIV-1; they were identified with immunoelectron microscopy and molecular sequencing as being HK2 [76,87]. Upregulation of HK2 expression has been shown to cause an immune response [88–93]. However, the disease spectrum where HK2 is upregulated has not been systematically investigated with a rigorous epidemiological design.

### (d) *In vivo* infectivity of HK2 is still under investigation

While the evidence of HK2 plasma RNA is robust, suggesting upregulation in certain diseases, there is no definitive evidence for the production of infectious particles. Infectious progenitors of HK2 have been constructed *in vitro* using existing HK2 DNA fragments from the human genome, providing proof of principle of the potential for HK2 infectious particle formation in humans today [31]. Furthermore, pseudo-typed viral particles with the HK2 *env* from locus K108 have been shown to be infectious in cell lines [32,94]. Isolation of sequences from patients and comparison of their mutations with copies in the published human genome have indicated purifying selection, which could result from copying of loci within the individual [87]. However, HK2 is insertionally polymorphic within the human population and many loci are not in the published human genome [28]. Therefore, the identification of new loci under purifying selection in contrast to the published loci is not conclusive proof of infectious activity. A more rigorous experimental design powered by high-throughput sequencing technologies and state-of-the-art molecular evolution analyses would provide a solid answer to the infectious potential of HK2.

### (e) Upregulation of HK2 could provide mobility/infectiousness of HERV-K

No HK2 locus has been found to be replication competent. However, there are HK2 proviruses with intact ORFs [27,30,33–35], and in mice, recombination between replication-defective ERV loci can lead to replication-competent loci [95,96]. This provides a plausible model for the reconstitution of mobile and/or infectious HK2 within the genome [97]. A recent paradigm of reconstitution of a replication competent ERV from two defective endogenous loci is the accidental laboratory recombination of defective endogenous murine leukaemia viruses (MLVs) which resulted in the xenotropic MLV-related retrovirus [95]. Young *et al*. [96] have recently shown the resurrection of defective ERVs in antibody-deficient mice through recombination, as a result of the upregulation of these ERVs by inflammation induced from microbiota in the gut. More importantly, we know that infectious HK2 can be reconstituted *in vitro* by the consensus of common HK2 loci within the genome [31,32], suggesting that, in principle, naturally occurring recombination could restore replication and infectivity.

## 7. Molecular basis of a possible HK2 connection with the development of cancer

HK2 loci produce one of two non-standard retroviral proteins, Np9 and Rec through alternatively spliced mRNA [81,98]. Rec is analogous to the Rev protein of HIV-1 and when highly expressed has been shown to promote tumour development in mice [99,100]. A region of the transmembrane domain of *env* has been found to be immunosuppressive in several retroviruses—the so-called immunosuppressive domain [101–103]. Heidmann and co-workers showed that tumours expressing *env* proteins of mouse mammary tumour virus can escape the immune response (at least transiently), whereas tumours having knocked-out *env* genes were efficiently recognized by the immune system and rejected successfully [104]. It must be noted, however, that evidence for immunosuppressive properties of the *env* of HK2 is scarce [105]. The emergence of replication competent/retrotransposing HK2 could be implicated in pathogenesis through insertional mutagenesis (extensively reviewed in [106]), a process which has been recently shown to be common for *L*1 and *Alu* retrotransposons [50]. Finally, another possible pathogenic mechanism would be through promoter activity of LTRs; for example, de-repression of one of the oldest HERVs has been recently connected with the development of Hodgkin's lymphoma [107].

The expression of oncogenic and immunosuppressive proteins by HK2 could contribute to the development of the malignant phenotype, complementing and enhancing the other numerous well-characterized factors (e.g. oncogenes). Because evolution of a cell towards the cancerous phenotype is a multi-stage, multi-factorial process, HK2 could be one of the many components enhancing the development of the selfish phenotype by increasing the aggressiveness or immune-stealth [108] property of the cell [109–111].

**Figure 3.**
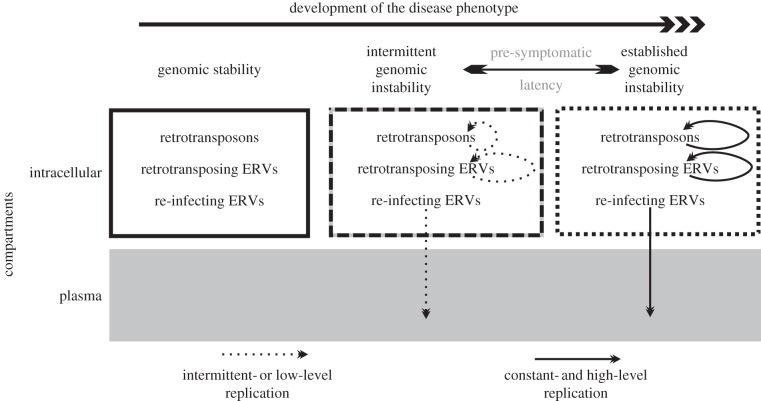
Model of ERV upregulation with respect to disease. HK2 RNA has been isolated from the plasma of patients with lymphoma and breast cancer. It is thought that upregulation of HK2 in these diseases could be connected with genomic instability which, however, affects most of the mobile elements. HK2 is isolated from the plasma because it is a re-infecting ERV and can form retrovirus-like particles.

Perhaps the upregulation of HK2 expression, independently of any HK2 activity in the early stages of tumourigenesis, provides the conditions for further involvement of HK2 in the latter stages of the development of the malignant phenotype. Very few apparently healthy individuals have detectable HK2 in the plasma [85]. This could be due to an inability to suppress ERV and retrotransposon expression/replication [112], which happens at an early stage throughout the development of the full disease phenotype. Quasi-defective antiviral (e.g. TRIM5*α* [113–115], RNAse-L [116,117]), silencing mechanisms or predisposition to genomic instability could allow upregulation/replication of HK2 well before the full development of the disease phenotype (pre-symptomatic latency; figure 3). An epidemiological study to define how widespread and early this phenomenon is, and what the significant underlying confounders are, could elucidate the mechanism associating HK2 with cancer.

## 8. The immune response against the upregulation of HK2

ERV antigens are host antigens and believed not to promote immune response upon their expression [118]. However, if an ERV antigen was not presented during the development of the immune system, then its later expression could promote an immune response [118]. Immune responses against HK2 antigens have been reported in patients with upregulated HK2 [119]. Nixon's group showed that stronger T-cell responses against HK2 antigens are related to slower progression to AIDS [88–91]. Some evidence suggests that this is not the consequence of immune deterioration in cases progressing to AIDS, but is rather the result of the immune system being tolerant to HERV antigens (not able to promote HERV-specific immune response). This group has also shown recently that CD8 T-cell responses against HK2 antigens resulted in the elimination of HIV-1 infected cells as a result of the upregulation of HK2 by HIV infections; this suggests that a HERV-based vaccine to provide long-term control of HIV might be possible [88]. Given that immunosuppression increases the incidence of specific cancers, a study to describe immune escape of HK2 as a result of generalized immunosuppression or HK2-specific immunotolerance with respect to the development of cancer, could provide insights on the immune escape of malignant cells [92,120].

## 9. Technical challenges in the study of HK2 mobility

As we have already noted, our ability to study TEs has greatly improved during the past five years. This is firstly due to the availability of a high-quality human reference genome [3] and secondly due to the development of higher throughput, lower-cost sequencing technologies. A HERV that is able to replicate within the human genome has never been described so far; determining whether HK2 is mobile within the genome could have potential clinical implications. To show mobility, we need to see insertional polymorphism within the same individual, i.e. we have to identify somatic integrations as loci present in a tissue but not in another tissue and exclude the possibility of deletion. Somatic integrations are expected to be random; therefore unless novel HK2 integrations are followed by clonal expansion of the cells, the recovery of a single locus against thousands of germline (i.e. within every cell) copies is a major experimental and bioinformatics challenge. Let us assume that we extract the genome of 1000 cells within which there have been 100 random novel somatic integrations in total. There are approximately 90 full-length HK2 loci in the human genome [26], so for the sake of simplicity, we will consider there to be 100. Assuming that solo LTRs (i.e. the naturally occurring product of recombinational deletion of full-length loci; figure 2) are 10 times more frequent [121], we expect that each one of the 100 novel integrations would have to be identified against a background of 1100 loci, each one having 1000 copies, in total 1 100 000 standard fixed HK2 copies. The problem becomes even more difficult if we consider 5000–6000 copies of SVAs, active transposons carrying a truncated LTR of HK2 [122]. Current high-throughput sequencing approaches are not expected to recover novel somatic integrations (though some researchers have been able to recover rare integrations [50,52]), unless there is a significant favourable bias for the novel integrations either owing to massive proliferation of the cell carrying the novel HK2 locus or through a novel experimental protocol which would enrich the novel and deplete the fixed HK2 loci. With respect to the bioinformatics challenge, the difficulty of mapping the high-throughput sequencing reads uniquely and identifying breakpoints is compounded by the fact that at least 50% of the human genome comprises repetitive regions. Longer, better and more reads per locus would help to establish which loci could be uniquely mapped within highly repetitive elements. We expect that the recent and forthcoming updates of high-throughput sequencing technologies will be able to dramatically improve this within 2013.

## 10. *Env* genes and their role in human disease

HK2 is the only HERV family that could be pathogenic through replication competent loci. On the other hand, replication-defective HERVs can contribute in pathophysiological mechanisms via their gene products. Here, we focus on the role of HERV *env* (envelope) genes and especially their immunosuppressive properties with respect to recent evolutionary insights pertaining to the mechanism of immune repression. Retroviral *env* genes have also been implicated in carcinogenesis as growth stimulators [123,124], but we shall not refer to this mechanism.

### (a) The burden of *env* genes

We have recently shown that the number of ERV loci generated following the initial germline invasion of a mammalian host by the virus is strongly inversely correlated to the integrity of the *env* gene, with the largest ERV families generally being derived from elements with deleted (or non-functional) envelopes [48]. The main way for a locus to produce many copies in the genome and be active in the long term is to lose its *env* gene and become a retrotransposon. There appears to be a trade-off between the integrity of the *env* gene and stable parasite–host coexistence, with the cost here being the loss of the ability to move between hosts. ERVs can be placed on a spectrum, with elements with intact *env* being the least adapted to intracellular life, and ERVs with no envelope at all being the most adapted to stable coexistence with the host.

In addition to being crucial for retroviral reinfection [12,13], several *env* genes have been independently co-opted by their mammalian hosts throughout their evolution, and contribute to two immune-related functions (other envelopes are involved in antiviral immunity [125]): development of the syncytiotrophoblast and immunotolerance of the mother to the paternal antigens of the fetus [126]. In humans, we are aware of two co-opted retroviral *env* genes, *syncytin*-1 and *syncytin*-2, both efficiently expressed at the syncytiotrophoblast [23,24]. In what follows, we bring two thought-provoking research questions about the role of *env* genes in cancer and autoimmunity.

### (b) Is immune escape of transmissible cancer related to the evolutionary development of the trophoblast and the co-option of *env* genes?

Causality in cancer has been dogmatically connected with acquired mutations (and replicating HK2 elements can be considered simply as mutations that could lead to cancer). A striking exception to this paradigm is transmissible cancer (as distinct from cancer associated with viral infection), of which two examples are known: canine transmissible venereal tumour and the Tasmanian devil facial tumour. In both cases, the genomes of neoplasms from different hosts are clearly more related to each other than to the genomes of the hosts in which they are found [127–129]. Therefore, it is believed that these tumours are serially transplanted between individuals, causing epidemics in dogs and Tasmanian devils.

The most exotic characteristic of transmissible cancer is that the infected individual's immune system fails to identify the transplanted cancer cells as foreign tissue (immune-tolerance to alloantigens) constituting a natural instance of allogeneic transplantation [129]. Transmission of cancer in humans has been observed as a rare phenomenon, predominantly as a side-effect of medically driven allogeneic tissue transplantation (usually followed by extensive immunosuppression to protect the allogeneic transplant from rejection) [130]. These rare cases support the policy of preventing individuals with cancer from donating their organs unless the primary tumour is within the central nervous system (where the probability of distant metastases is minimal and therefore the probability of cancer transplantation is negligible) [131]. Apart from the scarce medical cases of accidental cancer transplantation, there is an infrequent but well-described paradigm of naturally occurring allogeneic tumours in humans: gestational trophoblastic disease (GTD).

GTD occurs when a potentially malignant tumour, comprising a mixture of self and alloantigens, arises from the trophoblast of the placenta, during or after pregnancy [132]. The trophoblastic tumour develops by taking advantage of the naturally occurring immune-stealth cloak that placental mammals were forced to evolve to protect the embryo developing in the uterus, hiding and tolerating the fetus's alloantigens [133]. This immunotolerance is so efficient that it can sustain a totally allogeneic embryo as occurs in gestational surrogacy [134]. Apart from the well-developed immune-stealth cloak, GTD takes advantage of another two naturally occurring characteristics of the syncytiotrophoblast: tissue penetration and vascular remodelling [135]. The efficiency of these properties can be seen in ectopic pregnancy where the syncytiotrophoblast is accidentally implanted outside the uterus [136]. In such cases, the syncytiotrophoblast efficiently attaches to almost any tissue in the abdomen (e.g. intestines, peritoneum, spleen, liver, diaphragm), enhances circulation through vascular remodelling and then cannot be separated from the attached organs without extensive bleeding. Dramatically, the treatment of abdominal pregnancy is an operation where the surgeon is forced to concurrently remove the underlying tissues, a situation very similar to the removal of abdominal malignant tumours [137]. The adjuvant treatment is performed with methotrexate, a widely known antineoplastic substance [138]. It therefore seems that placental mammals have co-opted three crucial malignant attributes as a cost for well-protected offspring delivery: immune-tolerance of alloantigens, tissue penetration and vascular remodelling.

Heidmann and co-workers [104] have shown that it is possible to transplant allogeneic cancerous cell-lines in immunocompetent mice by engineering cancerous cells to express retroviral Env proteins. As discussed above, these genes are known to have been co-opted independently multiple times within the placental mammal lineage, where they are expressed in the placenta and play a crucial role for the normal formation of the syncytiotrophoblast [23,135,139–141]. While not all retroviral *env* genes have been co-opted for their immunosuppressive features (the most striking property of which is the promotion of cell fusion; hence they are called *syncytins*), their contribution to the immune-stealth cloak of the trophoblast is considered crucial for gestational immune tolerance [126]. It is thus reasonable to hypothesize that ectopic upregulation of immunosuppressive *syncytin* might contribute to the immune escape of human cancers (other than GTD).

Apart from showing the contribution of *syncytins* to the syncytiotrophoblast and thus possibly to allogeneic cancers in humans, Heidmann's experiments show a remarkable similarity in natural history to the canine transmissible venereal tumour: the *env*-expressing tumours grow, plateau and then regress [104,129]. This is in striking contrast to what happens in the Tasmanian devil, where the transmissible cancer is more aggressive, leading to a deadly and generalized disease [129]. This difference might be because the Tasmanian devil is a marsupial and is not likely to have developed a regulatory system for trophoblast-like activity. In addition, a possible insight into the regulation mechanisms in placental mammals comes from recent studies on repeated miscarriages in humans and the rapidly emerging field of clinical reproductive immunology [142]. While still considered too controversial to form a standard treatment guideline [143,144], it seems likely that a natural killer (NK) cell response in humans does not allow normal implantation of the placenta, resulting in recurrent miscarriages at least in some patients [145]. Interestingly, NK responses against the facial tumour could not be induced in Tasmanian devils after active immunization with facial cancer cells [146].

We could test the hypothesis for an *env-*induced immune-stealth cloak in transmissible cancers by implementing the methods used to discover and characterize *syncytins* [23,24,139–141]: *in silico* search for co-opted (or recently introduced) retroviral *env* genes within the tumour genomes, and *in vitro* verification of expressed and upregulated Env proteins in the tumour. The immunosuppressive properties of the Env protein could be verified in the same way as for the other *env* genes discussed previously [104].

### (c) Is *env* upregulation in autoimmunity a last resort for immune regulation?

The upregulation of HERVs has been described in a variety of diseases considered to be autoimmune, such as multiple sclerosis, rheumatoid arthritis and systemic lupus erythematosus [147–150]. The proposed underlying mechanism of association is that upregulated HERV-derived antigens are triggering an immune response that either attacks the antigen-producing cells, or cells producing similar antigens (molecular mimicry) [151], or that truncated *env* genes act as superantigens that trigger a non-specific autoimmune response [152]. While each of these hypotheses could be valid, the difficulty in proving such a mechanism is substantial. A significant correlation between the immune response against ERV antigens and autoimmune disease has been documented, although not always consistently found [153], but this correlation does not prove that ERV antigens were the trigger for disease onset. It might simply result from a general disruption of immune tolerance. The problem is further complicated by the fact that even for well-established auto-antibodies, the specificity remains low in the vast majority of diseases [154]. For example, there are a wide variety of auto-antibodies (e.g. anti-nuclear, anti-dsDNA) produced in inflammatory diseases, some of which are more commonly detected in older people with no clinical autoimmunity [155]. Thus, distinguishing cause from effect with respect to the role of HERVs and autoimmune diseases remains an open challenge.

On the other hand, HERVs could be involved in autoimmune disorders as a last resort for immune regulation if cells exploit immunosuppressive *env* antigens, such as HERV-H [19] and *syncytin*-2 [126]. Both these *env* antigens have been shown to be able to protect against rejection of allografts in mice [19,126]; therefore, the cells that produce these antigens could escape the irregular and prolonged immune responses which are the hallmark of autoimmunity. We propose that at least a proportion of the population of endogenous *env* genes (some of them having ORFs in the human genome [156]) could serve as a downregulatory mechanism of immunity and act as a population of expendable loci with common action rather than a single locus. Thus, although Env is among the viral proteins that may trigger, or be a target of, immune responses, these same proteins could serve an anti-autoimmune role.

This scenario could also explain the co-option of *env,* a major evolutionary paradox, as placentation exists throughout eutherian mammals, yet at the same time, the crucial *syncytin* gene has been derived independently multiple times from different ERV families to serve a similar purpose, presenting a very extreme example of convergent evolution [23,24,135,139–141,157,158]. Eutherian mammals have gradually acquired an array of innate immune genes (*tetherin*, TRIM5*α*, APOBEC, etc.) [125,159] in an evolutionary arms race between the host and its pathogens. If cell-mediated immune responses developed similarly, then the requirement of regulatory mechanisms against cell immunity would have increased. The population of endogenized *env* genes, some of them having immunosuppressive properties, could have provided the pool from which a true immunoregulatory gene could be selected. Eventually, one *env* gene prevailed and was co-opted to serve a specific immunoregulatory purpose (tolerance to fetal antigens), whereas the rest continued to degrade naturally (as described in §3) but were still able to provide immunosuppressive protection in extreme conditions. Intriguingly, we have never been able to identify an *env* gene for ERV-L, the oldest ERV family that we know within mammals. This is in line with our hypothesis: if, at the time of ERV-L's invasion, the cost of carrying *env* genes was not counterbalanced by their potential benefits in immunotolerance, this would lead to faster extinction of *env*-carrying families; more recently, as cell immunity became more intensive, *env* genes became useful at least transiently and eventually some of them became co-opted. Testing for recently mobile HERVs might be the most direct way to examine their association with cancer, but perhaps ancient integrations also contain clues for the underlying processes and could still remain as participants in certain present-day cancers.
